# Vitamin D deficiency and neuropathic pain in chronic spinal cord injury: a cross-sectional study

**DOI:** 10.3389/fnut.2025.1706735

**Published:** 2025-11-17

**Authors:** Tengbin Shi, Yanfeng He, Zhengxi Yu, Jiandong Li

**Affiliations:** 1 Department of Orthopedics, Fujian Medical University Union Hospital, Fuzhou, China; 2 School of Health, Fujian Medical University, Fuzhou, China; 3 Department of Minimally Invasive Spinal Surgery, the Affiliated Hospital of Putian University, Putian, China

**Keywords:** spinal cord injury, neuropathic pain, vitamin D deficiency, pain measurement, chronic pain, rehabilitation

## Abstract

**Background:**

Spinal cord injury (SCI) often leads to neuropathic pain (NeP), severely impacting patients’ function and rehabilitation. Vitamin D deficiency, highly prevalent in SCI due to reduced sun exposure and mobility, has been implicated in NeP in other conditions, but its role in chronic SCI-related NeP remains unclear.

**Methods:**

This cross-sectional study enrolled 182 adults with chronic traumatic SCI (≥6 month post-injury) currently admitted to 2 rehabilitation centers. Vitamin D status {serum 25-hydroxyvitamin D [25(OH)D]} was measured via high-performance liquid chromatography–tandem mass spectrometry (HPLC-MS/MS). NeP was diagnosed using the Douleur Neuropathique 4 (DN4) questionnaire (score ≥4/10) in combination with clinical assessment by a physician. Multivariate logistic regression, controlling for confounders (e.g., age, comorbidity, Charlson Comorbidity Index, CCI), injury severity (American Spinal Injury Association Impairment Scale, and AIS grade), assessed the association between vitamin D levels and NeP. Receiver operating characteristic (ROC) curve analysis was performed to identify a predictive cutoff.

**Results:**

The prevalence of NeP was 52.7% (96/182). Vitamin D deficiency or insufficiency [25(OH)D < 30 ng/mL] affected 64.8% (118/182) of participants. Lower vitamin D levels were strongly and independently associated with NeP risk. Individuals in the lowest vitamin D tertile (2.00–11.68 ng/mL) had significantly higher adjusted odds of NeP compared to those in the highest tertile (16.71–23.03 ng/mL) [adjusted odds ratio: 4.8, 95% CI: (3.4, 6.8), *p* < 0.001]. ROC analysis identified a serum 25(OH)D level <16.35 ng/mL as a predictive cutoff for NeP (area under the curve = 0.812, sensitivity 79.1%, specificity 71.9%).

**Conclusion:**

Vitamin D deficiency is highly prevalent and a strong, independent predictor of neuropathic pain in individuals with chronic SCI. These cross-sectional findings suggest routine screening for vitamin D deficiency might be indicated; however, interventional trials are needed to confirm a potential therapeutic role of vitamin D supplementation in managing SCI-related NeP.

## Introduction

Beyond sensory and motor dysfunction, spinal cord injury (SCI) frequently involves neuropathic pain (NeP), with an overall point prevalence of 53% (1). Recent data indicate that 30–50% of individuals with SCI develop NeP within 1 year post-injury—significantly exceeding rates in the general population (7–10%) (2, 3). Distinct from nociceptive pain, NeP typically arises without identifiable external stimuli due to aberrant nervous system signaling. It manifests clinically as spontaneous pain, hypoesthesia, allodynia, and sensory deficits (4–6). Beyond severely impairing daily function and psychological wellbeing, NeP correlates strongly with anxiety, depression, and sleep disturbances, while complicating and impeding rehabilitation efforts (1, 3, 5–7).

Currently, the treatment of neuropathic pain following spinal cord injury (SCI) remains a pressing clinical challenge. Current therapeutic approaches include first-line pharmacotherapies—such as anticonvulsants (pregabalin, gabapentin), serotonin-norepinephrine reuptake inhibitors (SNRIs), and tricyclic antidepressants (e.g., amitriptyline)—alongside physical modalities such as transcranial direct current stimulation (tDCS), transcutaneous electrical nerve stimulation, and psychological interventions. Nevertheless, these treatments frequently yield suboptimal outcomes, with limited robust evidence supporting their efficacy (8, 9). The development of novel analgesic strategies is hindered by the complex, poorly understood pathophysiology of SCI-related NeP, which involves distinct mechanisms from other neuropathic conditions. Furthermore, the multidimensional sequelae of SCI itself suggest that factors driving NeP in this population may differ fundamentally from those in other diseases. Consequently, a dedicated investigation into SCI-specific NeP mechanisms is critical for identifying targeted therapeutic approaches and improving patient outcomes.

Vitamin D, a fat-soluble vitamin primarily existing as cholecalciferol (D3) and ergocalciferol (D2), is classically recognized for regulating calcium metabolism and bone homeostasis. Emerging evidence now underscores its significant—yet underappreciated—role in nervous system function and pain modulation (10–13). Vitamin D deficiency {commonly defined as serum 25-hydroxyvitamin D [25(OH)D] < 20 ng/mL or <50 nmol/L} and insufficiency (20–29 ng/mL or 50–74 nmol/L) represent a major global public health concern (14, 15). As a neuroactive steroid, vitamin D deficiency is implicated in disrupted nociception and neuromuscular dysfunction among individuals with chronic pain (16, 17). This deficit is particularly prevalent in neurological populations, with significantly higher rates observed in traumatic brain injury (18), stroke (19), or SCI cohorts (20, 21) compared to healthy individuals, often attributable to factors such as reduced sun exposure and mobility.

Evidence indicates that vitamin D deficiency significantly contributes to neuropathic pain in diabetic peripheral neuropathy, cardiovascular autonomic neuropathy, post-herpetic neuralgia, and rheumatoid arthritis (22–24). Vitamin D supplementation may serve as an effective adjunctive therapy for such conditions. Animal studies further demonstrate that long-term vitamin D administration reduces neuropathic pain behavioral scores in rats, potentially through modulation of pro-oxidant/antioxidant markers in the spinal cord (25). Chronically deficient states may also compromise immune function and promote persistent inflammation (26). Nevertheless, the role of vitamin D deficiency in neuropathic pain following chronic spinal cord injury (SCI) remains inadequately investigated. Given the high prevalence of vitamin D deficiency in SCI populations—driven by reduced sun exposure, immobilization, and impaired renal function—elucidating its relationship with neuropathic pain severity holds critical clinical relevance.

Therefore, this cross-sectional study aims to evaluate the prevalence of vitamin D deficiency and insufficiency among individuals with chronic SCI and to explore the association between vitamin D levels and the presence of neuropathic pain. It is hypothesized that vitamin D deficiency is highly prevalent and is associated with a higher risk of NeP in this population. The findings may help to identify a potentially modifiable risk factor and inform future research into nutritional interventions aimed at improving pain management and quality of life.

## Methods

### Study design and participants

This cross-sectional study was conducted and reported in accordance with the Strengthening the Reporting of Observational Studies in Epidemiology (STROBE) guidelines. The study retrospectively enrolled consecutive adults (aged 18–85 years) with chronic traumatic SCI (injury duration ≥6 months; neurological levels C2–L2; AIS grades A–D) who were admitted to two tertiary rehabilitation centers specializing in acute traumatic SCI between January 2018 and February 2024. Ethical approval was granted by the local institutional review board with a waiver of informed consent. Exclusion criteria included individuals with (1) acute spinal cord injury; (2) comorbid conditions affecting pain perception peripheral neuropathy, diabetes mellitus, malignancy, or severe spasticity (Ashworth grade 3 or higher); (3) current use of vitamin D supplements exceeding standard recommendations (>10,000 IU/day); (4) disorders altering vitamin D metabolism (e.g., hyperparathyroidism and renal failure); or (5) significant psychological comorbidities (e.g., major depressive disorder and severe anxiety) documented in their medical record that could significantly influence pain perception or reporting.

### Data collection and assessments

Demographic characteristics (age, sex, BMI, smoking/alcohol status, and level of education), medical history [including Charlson Comorbidity Index (CCI)], and SCI-specific variables (injury level, etiology, time since injury, American Spinal Injury Association (ASIA) Impairment Scale (AIS) grade, Injury Severity Score [ISS], and surgical intervention/timing) were systematically recorded in electronic medical records. The intake of vitamin D supplements (yes/no and dosage, if available) was specifically assessed and recorded. Medication intake relevant to pain modulation (e.g., anticonvulsants, antidepressants, and analgesics) was also documented. Pain characteristics documented included: severity, assessed via the Numeric Rating Scale (NRS) and the Short-Form McGill Pain Questionnaire (SF-MPQ); location, marked by participants on a body diagram; and nature, classified according to the International Spinal Cord Injury Pain (ISCIP) classification. Exacerbating/relieving factors, functional impact spinal cord independence measure (SCIM) (27), and current pain treatments were also recorded. All data, including baseline serum vitamin D levels (the first available measurement post-admission), were extracted from medical records at a uniform timepoint for all participants, defined as the time of their initial comprehensive rehabilitation assessment conducted within the first 2 weeks following admission.

### Neurological and pain assessments

Neurological status was evaluated using the *International Standards for Neurological Classification of Spinal Cord Injury* (ISNCSCI) (28) to determine injury level and AIS grade.

In this study, NeP following SCI was defined based on a combination of the following criteria: 1. pain localized at/below the injury level; 2. a score of ≥4 on the Douleur Neuropathique 4 (DN4) questionnaire (Chinese version); and 3. corroborating sensory abnormalities (e.g., hypoesthesia and allodynia) identified on the sensory examination component of the ISNCSCI. The DN4 is a 10-item questionnaire based on sensory descriptions and examinations, with a specificity of 82.9% and a sensitivity of 89.9% for detecting neuropathic pain (29) assessed: Patient-reported descriptors (seven items: burning, cold, electric shocks, tingling, pins/needles, numbness, and itching); and bedside sensory findings (three items: hypoesthesia, pinprick hypoalgesia, and dynamic allodynia). To assess the locations of pain, participants used a body diagram to mark the areas where they experienced the most pain. The pain was classified according to the ISCIP classification (4), a mechanism-based classification that distinguishes between nociceptive pain, neuropathic pain, other pain, and unknown pain in patients with SCI, which has been validated by ASIA and the International Spinal Cord Society (ISCoS).

All assessments, including the DN4 and neurological examinations (30), were performed independently by trained physicians, with discrepancies resolved via consensus or senior clinician consultation.

### Vitamin D quantification

Fasting venous blood samples were collected in EDTA tubes, centrifuged (1,459 × g, 15 min, 4 °C), and stored at −80 °C. Total serum 25-hydroxyvitamin D [25(OH)D] was measured via HPLC-MS/MS (sum of 25(OH)D₂ and 25(OH)D₃), with: The assay is linear up to 100 ng/mL, and sensitive to 1 ng/mL. Day-to-day precision (%CV) at various levels of 25(OH)D ranged from 5.6 to 8.5%. Vitamin D status was categorized per the Endocrine Society clinical practice guidelines (31) as follows: deficiency [25(OH)D < 20 ng/mL], insufficiency [25(OH)D < 30 ng/mL], and sufficiency [25(OH)D ≥ 30 ng/mL].

### Statistical analysis

Data were presented as mean ± standard deviation (SD) (or median with interquartile range (IQR), where applicable) for continuous variables and frequencies for categorical variables. Normality was tested with the Shapiro–Wilk test. Group comparisons used the Mann–Whitney U-test for non-parametric variables, Student’s *t*-test for parametric variables, and the χ^2^ test for categorical variables. Risk factors for neuropathic pain were investigated by univariate regression analysis, and the results were controlled using a binary logistic regression model. Binary logistic regression analysis (forward stepwise LR method) was used to control the results. Variables that exhibited statistical significance (*p* < 0.1) in the univariate regression analysis or were deemed clinically relevant were included in the multivariate analysis. The Box–Tidwell test was applied to evaluate the assumption of linearity in the logit for continuous variables. Multicollinearity was assessed using the variance inflation factor in a multiple regression model with the same variables. Results are reported as odds ratios (ORs) with 95% CIs (confidence intervals). The sample size in our study met the demands of events per variable during logistic regression analysis. Receiver operating characteristic (ROC) curve analysis was performed to determine the predictive capability of serum 25(OH)D levels for NeP and to identify an optimal cutoff value (by maximizing the Youden index), reported with the area under the curve (AUC), sensitivity, and specificity. To verify the robustness of the identified serum 25(OH)D cutoff value for NeP, we performed sensitivity analyses including: (1) an internal validation using 1,000 bootstrap replicates to assess the stability of the cutoff and its associated performance metrics and (2) recalculation of the cutoff using alternative criteria, including minimizing the distance to the top-left corner of the ROC curve and prioritizing high sensitivity (≥90%). Furthermore, vitamin D levels were stratified into tertiles to explore a dose–response relationship. The handling of missing data was explicitly addressed; cases with missing values for key variables (e.g., vitamin D level and DN4 score) were excluded from the respective analyses, and the amount of missing data for each variable is reported. A two-tailed *p* < 0.05 was considered statistically significant. All analyses were performed using SPSS Statistics version 26.0 (IBM Corp., Armonk, NY, United States) and GraphPad Prism version 8 (GraphPad Software, San Diego, CA, United States).

## Results

**Figure 1:**
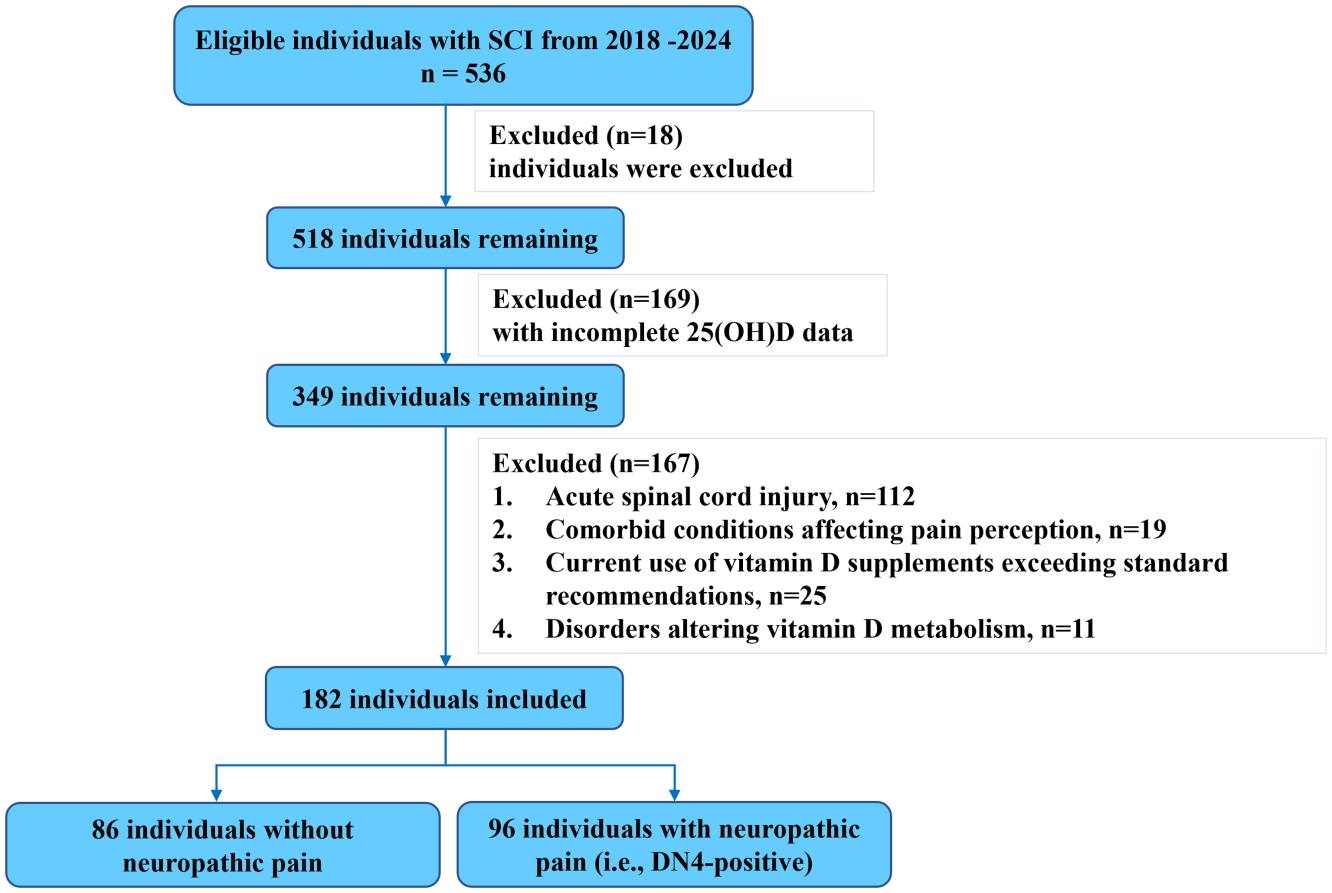
Flow diagram of the study.

The general demographic and clinical characteristics of the participants are summarized in Table 1 (see Figure 1 for participant flow). The cohort had a mean age of 58.2 ± 13.2 years and was predominantly male (138/182, 75.8%). The primary mechanisms of injury were falls (97/182, 53.3%) and traffic accidents (65/182, 35.7%). Regarding the neurological level of injury (NLI), cervical spine injuries constituted 68.1% (124/182), with C1-4 injury levels accounting for 87 cases (47.8%) and C7-T1 injury levels accounting for 37 cases (20.3%). Thoracic level injuries (T2-T10) were present in 20 individuals (11.0%), and thoracolumbar injuries (T11-L2) in 38 individuals (20.9%). According to the AIS grade, the distribution was as follows: 64 individuals (35.2%) were grade A, 26 (14.3%) grade B, 44 (24.2%) grade C, and 48 (26.4%) grade D. Reflecting the acuity and severity of the conditions leading to their current inpatient rehabilitation admission, 42 participants (23.1%) had a history of tracheotomy, and 131 participants (72.0%) had undergone decompression and/or fixation surgery within 24 h of their initial injury. Notably, a high prevalence of suboptimal vitamin D status was observed, with 64.8% (118/182) of participants having 25(OH)D levels <30 ng/mL.

**Table 1:** Characteristics of the 182 individuals with spinal cord injury included in the analyses.

Characteristics	Mean [SD/(IQR)] or N (%)
Age (y)	58.2 (13.2)
Sex	
Female	44 (24.2%)
Male	138 (75.8%)
Time post injury (y) (median)	6.6 (5.6)
Body mass index (kg/m^2^)	
<24	75 (41.2%)
≥24	107 (58.8%)
Diabetes	26 (14.3%)
Current smoker	45 (24.7%)
Alcohol consumers	51 (28.0%)
Charlson comorbidity index	0.00 [0.00; 2.00]
Vitamin D, ng/mL	21.8 (13.4)
Vitamin D status	
Deficient (<20 ng/mL)	117 (36.8%)
Insufficient	47 (28.0%)
Normal	18 (19.8%)
Highest level of education	
Illiterate	23 (12.6%)
Pre-high school	65 (35.7%)
High school	69 (37.9%)
College and above	25 (13.7%)
Etiology	
Falls	97 (53.3%)
Traffic accident	65 (35.7%)
Violence	11 (6.0%)
Others	9 (4.9%)
ISS	
<16	131 (72.0%)
≥16	51 (28.0%)
Neurological level of injury	
High cervical (C1–C4)	87 (47.8%)
Low cervical (C5–T1)	37 (20.3%)
Thoracic (T2–T10)	20 (11.0%)
Thoracolumbar (T11–L2)	38 (20.9%)
AIS grade	
A	64 (35.2%)
B	26 (14.3%)
C	44 (24.2%)
D	48 (26.4%)
Functional independence (SCIM score)	46 (26.3)
Treated with high-dose methylprednisolone	62 (34.1%)
History of tracheotomy	42 (23.1%)
Pain medication	77 (42.3%)
Time to admission, hours	5.47 [1.64; 113]
Surgery within 24 h	131 (72.0%)
Length of stay, days	67.7 [45.8; 121]
ICU stay	117 (64.3%)
Length of ICU stay, days	7.81 [2.50; 28]
Follow-up duration, months	39.5 (24.2)

SD, standard deviation; AIS, American Spinal Injury Association (ASIA) Impairment Scale; SCIM spinal cord independence measure. IQR, interquartile range; ICU, intensive care unit.

### Neuropathic pain prevalence and characteristics

Based on the pre-defined diagnostic criteria, NeP was identified in 52.7% (96/182) of the participants (Table 2). The locations of NeP were categorized as follows: at-level NeP (located at the neurological level of injury (NLI) or within three dermatomes below this level) was present in 51.0% (49/96) of those with NeP, below-level NeP (located more than three dermatomes below the NLI) was reported by 37.5% (36/96), and 11.5% (11/96) reported diffuse NeP that could not be definitively linked to the SCI (*note: percentages sum to >100% as some individuals reported pain in multiple categories*). Regarding the timing of NeP onset after injury, 11.5% (11/96) of individuals with NeP reported its emergence within the first 3 months post-injury, while the majority (88.5%, 85/96) developed it during the chronic phase (>3 months post-injury). The most frequently reported pain qualities among individuals with NeP were burning sensation (42.6%) and electric shock-like pain (28.9%).

**Table 2:** Comparison of individuals with (DN4 ≥ 4) and without neuropathic pain.

Characteristics	NeP + (*n* = 96)	NeP − (*n* **=** 86)	*P*-value
Age (y)	58.6 (13.5)	56.3 (16.9)	0.037^*^
Sex			0.625
Female	23 (24.0)	18 (20.9)	
Male	73 (76.0)	68 (79.1)	
Time post injury (y)	6.2 (5.5)	6.1 (5.6)	0.863
BMI (kg/m^2^)			0.462
<24	42(43.8)	33 (38.4)	
≥24	54 (56.3)	53 (61.6)	
Diabetes	17 (17.7)	9 (10.5)	0.163
Current smoker	25 (26.0)	20 (23.3)	0.664
Alcohol consumers	28 (29.2)	23 (26.7)	0.716
CCI	1.0 [0.55; 2.00]	3 [1.25; 3.50]	0.013
Vitamin D, ng/mL	13.7 (10.7)	22.7 (16.2)	<0.001
Vitamin D status			<0.001
Deficient (≤20 ng/mL)	82 (85.4)	35 (40.7)	
Insufficient (21–29 ng/mL)	12 (12.5)	35 (40.7)	
Normal (≥30 ng/mL)	2 (2.1)	16 (18.6)	
Highest level of education			0.246
Illiterate	8 (8.3)	15 (17.4)	
Pre–high school	34 (35.4)	31 (36.0)	
High school	37 (38.5)	32 (37.2)	
College and above	17 (17.7)	8 (9.3)	
Etiology			0.855
Falls	51 (53.1)	46 (53.5)	
Traffic accident	34 (35.4)	31 (36.0)	
Violence	7 (7.3)	4 (4.7)	
Others	4 (4.2)	5 (5.8)	
ISS			0.843
<16	50 (32.3)	61 (37.0)	
≥16	68 (43.9)	68 (41.2)	
Neurological level of injury			0.905
High cervical (C1–C4)	47 (49.0)	40 (46.5)	
Low cervical (C5–T1)	20 (20.8)	17 (19.8)	
Thoracic (T2–T10)	9 (9.4)	11 (12.8)	
Thoracolumbar (T11–L2)	20 (20.8)	18 (20.9)	
AIS grade			0.003^*^
A	42 (43.8)	22 (25.6)	
B	16 (16.7)	10 (11.6)	
C	23 (24.0)	21 (24.4)	
D	15 (15.6)	33 (38.4)	
Functional independence (SCIM score)	43 (16.2)	52 (14.8)	<0.01
Treated with high-dose methylprednisolone	46 (47.9)	35 (40.7)	0.328
History of tracheotomy	21 (21.9)	16 (18.6)	0.877
Surgery within 24 h	56 (58.3)	63 (73.3)	0.035
Pain medication	45 (46.9)	26 (30.2)	0.022
Time to admission, hours	4.87 [1.64; 113]	6.08 [2.34; 220]	0.645
Length of stay, days	63.6 [42.4; 121]	71.7 [43.1; 135]	0.037
ICU stay	65 (67.7)	52 (60.5)	0.309
Length of ICU stay, days	7.68 [2.18; 26]	7.81 [3.67; 41]	0.015
Neurogenic bladder and/or bowel	81 (84.4)	36 (41.9)	<0.001
Follow-up duration, months	36.3 (21.2)	38.4 (22.5)	0.766

Chi-square and independent samples t-test used, *p < 0.05; BMI, body mass index; CCI, Charlson comorbidity index; AIS, American Spinal Injury Association (ASIA) Impairment Scale; SCIM, spinal cord independence measure.

Comparisons between participants with and without NeP are detailed in Table 2. Briefly, individuals with NeP were significantly older, had a higher comorbidity burden (CCI), had less severe injuries, and had poorer functional independence (lower SCIM scores) compared to those without NeP. Significant differences were also observed in clinical management factors, including longer overall length of stay, longer ICU stays, and a higher prevalence of neurogenic bladder and bowel dysfunction (84.4% vs. 41.9% in those without NeP, *p* < 0.001). Furthermore, serum 25(OH)D levels were significantly lower in the NeP group. No significant differences were found between the groups regarding injury etiology, education level, time since injury, history of tracheotomy, treatment with high-dose methylprednisolone, smoking status, or alcohol use.

### Association between vitamin D levels and NeP

As shown in Table 3, potential predictors of NeP identified in univariate regression analysis included older age, higher CCI, lower serum 25(OH)D level, higher AIS grade, longer length of stay, longer length of ICU stay, and lower SCIM score. Multicollinearity effects were ruled out as the variance inflation factor (VIF) values for all independent variables were below 3, and the average VIF value for the entire regression model was 1.68.

**Table 3:** Putative predictors of neuropathic pain (DN4 ≥ 4): univariate and multivariable associations.

Characteristics	VIF value	Univariate regression		Multivariable regression	
		OR (95% CI)	*P*-value	OR (95%CI)	*P*-value
Age (y)	2.74	1.68 (1.542–1.835)	0.012	1.05 (0.992–1.084)	0.06
CCI	2.71	2.13 (1.133–1.364)	0.036	2.61 (1.651–2.633)	0.01
Vitamin D, ng/mL	1.88	0.81 (0.75–0.86)	<0.001	0.53 (0.392; 0.684)	<0.001
AIS grade	1.97		0.005		0.030
A	–	2.10 (2.453–4.480)	–	3.02 (2.197–4.153)	–
B	–	1.86 (1.892–3.368)	–	2.32 (1.698–3.159)	–
C	–	1.64 (1.402–2.498)	–	1.73 (1.278–2.348)	–
D	–	–	–	–	–
Functional independence (SCIM score)	1.65	0.98 (0.932–0.981)	0.017	0.898 (0.997–1.081)	0.711
Surgery within 24 h	1.42	0.89 (0.738–1.078)	0.512	1.006 (0.988–1.033)	0.181
Length of stay, days	1.87	1.08 (0.981–1.178)	0.01	1.02 (1.001–1.040)	0.37
Length of ICU stay, days	1.90	1.24 (1.133–1.364)	0.03	1.79 (1.024–1.315)	0.63
Pain medication	1.94	1.18 (1.018–1.493)	0.441	0.97 (0.931–1.095)	0.323

BMI body mass index; CCI Charlson comorbidity index; CI confidence interval; OR odds ratio; AIS, American Spinal Injury Association (ASIA) Impairment Scale; SCIM, spinal cord independence measure; VIF, variance inflation factor.

Variables with a univariate *p* < 0.1 and those deemed clinically relevant were entered into a multivariable binary logistic regression model using a forward stepwise selection process. The final model, adjusted for confounders, identified three factors as being significantly and independently associated with NeP: lower serum 25(OH)D level, a higher CCI score, and a higher AIS grade. There was a significant negative correlation between vitamin D levels and the presence of NeP (*p* < 0.001).

To further explore the dose–response relationship, vitamin D levels were stratified into tertiles (Table 4). After adjusting for confounders, compared to participants in the third tertile of vitamin D levels (16.71–23.03 ng/mL), those in the second tertile (11.69–16.70 ng/mL) had almost three times the adjusted odds of NeP, and those in the first tertile (2.00–11.68 ng/mL) had more than five times the adjusted odds.

**Table 4:** Risk of having neuropathic pain according to vitamin D levels: logistic regression analyses.

Vitamin D levels	OR (95% CI)	*p*-value
Reference (3rd tertile) 2nd tertile	2.6 (2.1; 4.9)	<0.001
Reference (third tertile) first tertile	4.8 (3.4; 6.8)	<0.001

Analyses were adjusted for: age, Charlson comorbidity index, and functional independence in activities of daily living (SCIM score). Vitamin D levels: first tertile = 2.00–11.68 ng/mL, second tertile = 11.69–16.70 ng/mL, and third tertile = 16.71–23.03 ng/mL.

**Figure 2:**
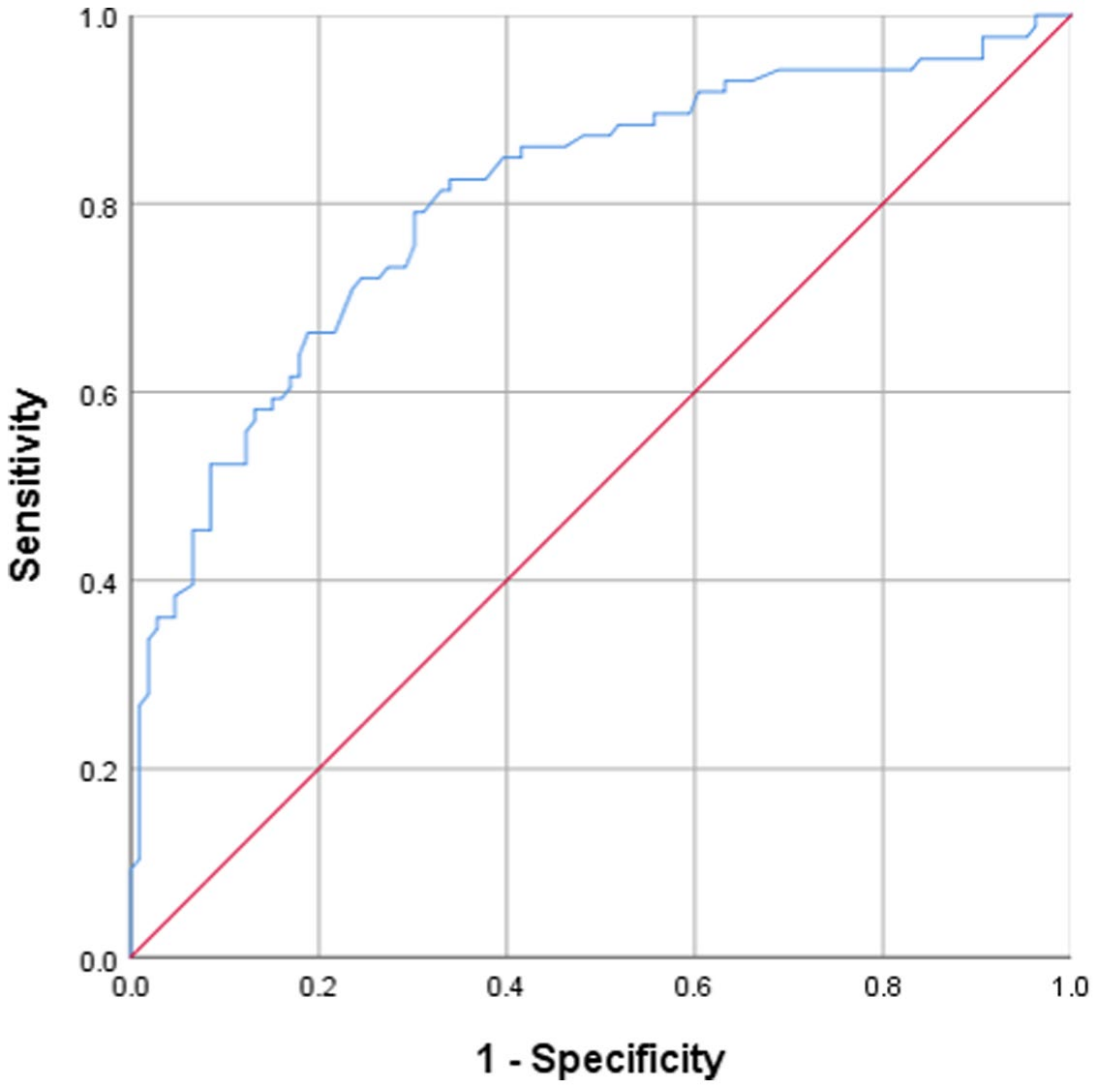
Receiver operating characteristics (ROC) analysis over 25(OH)D levels shows threshold values with specificity and sensitivity in predicting neuropathic pain, AUC = 0.812 [95% confidence interval (CI): 74.9–87.6; sensitivity = 79.1%; specificity = 71.9%] with a cutoff value of 16.35 ng/mL.

ROC curve analysis was performed to evaluate the predictive utility of serum 25(OH)D level for NeP and to identify a clinically applicable cutoff value. The analysis indicated that a 25(OH)D level <16.35 ng/mL could distinguish participants with NeP, with a specificity of 71.9% and a sensitivity of 79.1% (AUC: 81.2%; 95% CI: 74.9–87.6%) (Figure 2). Sensitivity analyses confirmed the robustness of the identified 25(OH)D cutoff (<16.35 ng/mL). Bootstrap internal validation (1,000 replicates) yielded a mean cutoff of 16.52 ng/mL (95% CI: 15.21–17.88), closely matching our original finding. The bootstrapped mean AUC was 0.805 (95% CI, 0.742–0.868). When applied to the bootstrap samples, the original cutoff maintained a mean sensitivity of 78.3% (95% CI: 70.5–85.2%) and specificity of 71.2% (95% CI: 62.8–78.9%). Overall, among the 81 participants with 25(OH)D levels below 16.35 ng/mL, 74 (91.4%) exhibited NeP, whereas only 32 (31.7%) of the 101 participants with levels ≥16.35 ng/mL had NeP (*p* < 0.0001).

## Discussion

This study provides a systematic evaluation of the association between serum vitamin D levels and NeP in individuals with chronic SCI. The primary finding of this cross-sectional investigation is the high observed prevalence of NeP in this inpatient rehabilitation cohort, reaching 52.7% (96/182), which aligns with established epidemiological data (1, 5, 32). Notably, the vast majority (88.5%) of NeP cases emerged during the chronic phase (>3 months post-injury), highlighting the persistent nature of this condition and the need for sustained pain management focus long after the initial injury. In our cohort, individuals with NeP presented with greater functional impairment (lower SCIM scores) and a higher comorbidity burden (elevated CCI). Contrary to some expectations, NeP was associated with less severe neurological injuries (higher AIS grades, i.e., more motor-incomplete injuries). Furthermore, neurogenic bladder/bowel dysfunction was significantly more prevalent in those with NeP (84.4%, 81/96) compared to those without (41.9%, 36/86, *p* < 0.001).

A substantial 64.8% (118/182) of patients had suboptimal vitamin D status [25(OH)D < 30 ng/mL]—a prevalence markedly higher than the 20–40% typically reported in the general population (3). This elevated rate likely reflects the cumulative effect of well-established risk factors in the SCI population, such as severely reduced mobility and sun exposure. The high prevalence of conditions requiring intensive initial care (e.g., 23.1% with a history of tracheotomy) may further limit outdoor activity early on, potentially exacerbating and prolonging deficiency.

Multivariate regression analysis, adjusting for relevant confounders, identified serum 25(OH)D level as a strong and independent factor associated with NeP (adjusted odds ratio (aOR) = 0.53 per ng/mL increase, 95% CI: 0.392–0.684, *p* < 0.001). To the best of our knowledge, this is the first study to report such an independent association in a chronic SCI population. A clear dose–response relationship was observed: compared to participants in the highest vitamin D tertile (16.71–23.03 ng/mL), the adjusted odds of NeP were approximately three times higher for those in the middle tertile (11.69–16.70 ng/mL) (aOR = 2.6, 95% CI: 2.1–4.9, *p* < 0.001) and more than five times higher for those in the lowest tertile (2.00–11.68 ng/mL) (aOR = 4.8, 95% CI: 3.4–6.8, *p* < 0.001). ROC curve analysis identified 25(OH)D < 16.35 ng/mL as a discriminative cutoff for NeP (AUC = 0.812; sensitivity 79.1%, specificity 71.9%). The clinical relevance of this threshold is underscored by the finding that 91.4% (74/81) of participants with 25(OH)D levels below this cutoff had NeP, compared to only 31.7% (32/101) of those with levels at or above it (*p* < 0.001). However, as this is cross-sectional data, this association does not imply causation and requires validation in longitudinal and interventional studies.

Preclinical models highlighted the neuroprotective effect of vitamin D, which is associated with enhancing axonal and neuronal survival, suppression of neuroinflammation, modulation of autophagy, and contributions to neurogenesis and synaptic plasticity (33, 34). These insights underscore the potential for vitamin D supplementation not only to decelerate the progression of neurodegenerative diseases but also to improve the quality of life in patients with chronic CNS injury disorders such as SCI. Vitamin D may exert its effects partly through the vitamin D receptor (VDR) expressed in microglia/macrophages. In a study by Cui et al. (35), a significant upregulation of VDR was observed in microglia/macrophages surrounding the infarct area following cerebral ischemia–reperfusion in mice. Conditional inactivation of VDR in microglia/macrophages markedly increased infarct volume and neurological deficits, accompanied by a robust proinflammatory phenotype characterized by elevated secretion of TNF-*α* and IFN-*γ*, as well as enhanced infiltration of peripheral T lymphocytes. Blockade of TNF-α and IFN-γ significantly ameliorated these outcomes, untangling a novel mechanism underlying the association between vitamin D deficiency and adverse outcomes in CNS injury. Furthermore, vitamin D receptors in dorsal root ganglia and spinal cord are known to modulate neurotrophic factors (e.g., BDNF and NT-3), influencing neuronal repair (36). Additionally, the transient receptor potential vanilloid 1 (TRPV1) channel, which plays a crucial role in nociception, inflammation, and immunity, is regulated by various lipophilic ligands (37). Evidence suggests that vitamin D can act as an endogenous regulator of the TRPV1 channel (38, 39), potentially representing another mechanism through which it might influence neuropathic pain. However, the precise relationship between these intricate mechanisms and the development of neuropathic pain specifically after SCI remains to be fully elucidated and constitutes an important avenue for future research.

In our study, serum 25(OH)D level remained independently associated with neuropathic pain after adjustment for comorbidity burden (CCI) and injury severity (AIS grade), suggesting that its role extends beyond these traditional risk factors. While our cross-sectional findings highlighted a strong and independent association, they cannot establish causality or the efficacy of treatment. Therefore, based on these results, we can only suggest that routine 25(OH)D screening *might be considered* in individuals with chronic SCI. If vitamin D deficiency is identified, supplementation according to established clinical guidelines could be initiated. Future prospective longitudinal studies are needed to confirm if low vitamin D is a risk factor for NeP development, and interventional trials are essential to definitively assess the impact of vitamin D repletion on pain outcomes in this population.

This study has several limitations. First, the cross-sectional design precludes any causal inferences regarding the relationship between vitamin D and NeP. While a strong, dose-dependent association was observed, the temporal sequence remains uncertain; it is plausible that NeP leads to reduced outdoor activity and consequently lower sun exposure, contributing to vitamin D deficiency. Prospective longitudinal and interventional studies are urgently needed to evaluate whether vitamin D supplementation can prevent or ameliorate NeP in individuals with SCI. Second, despite adjusting for available clinical and demographic variables, unmeasured factors such as dietary vitamin D intake, precise sun exposure duration, skin pigmentation, and use of non-prescription supplements were not systematically collected, which might confound the results. Third, the generalizability of our findings may be limited, as participants were recruited from two rehabilitation centers in China, and genetic, dietary, and climatic differences may limit their applicability to other populations. Furthermore, the retrospective data extraction and the exclusion of patients with comorbidities affecting vitamin D metabolism (e.g., chronic kidney disease) may introduce a potential for selection bias, potentially resulting in a cohort that is not fully representative of the broader SCI community. Finally, the lack of data on key vitamin D pathway components, such as polymorphisms in vitamin D metabolism genes or levels of the active metabolite 1,25-dihydroxyvitamin D₃ [1,25(OH)₂D₃], limits the depth of mechanistic insight.

In conclusion, this study of 182 chronic SCI patients revealed a high prevalence of neuropathic pain and vitamin D deficiency or insufficiency. Most notably, lower serum 25(OH)D levels were identified as a strong and independent factor associated with the presence of NeP, exhibiting a clear dose–response relationship. These cross-sectional findings suggest that hypovitaminosis D may be a significant and potentially modifiable risk factor in the complex pathophysiology of post-SCI pain. Based on these results, routine 25(OH)D screening might be considered in the management of chronic SCI. Ultimately, future prospective interventional studies are required to definitively investigate whether vitamin D supplementation reduces the incidence or severity of NeP and to elucidate the potential mechanistic links between vitamin D status and central sensitization processes following SCI.

## Data Availability

The original contributions presented in the study are included in the article/supplementary material, further inquiries can be directed to the corresponding authors.
